# Mixed-Method Quasi-Experimental Study of Outcomes of a Large-Scale Multilevel Economic and Food Security Intervention on HIV Vulnerability in Rural Malawi

**DOI:** 10.1007/s10461-016-1455-1

**Published:** 2016-06-27

**Authors:** Lance S Weinhardt, Loren W Galvao, Alice F Yan, Patricia Stevens, Thokozani Ng’ombe Mwenyekonde, Emmanuel Ngui, Lindsay Emer, Katarina M Grande, Lucy Mkandawire-Valhmu, Susan C Watkins

**Affiliations:** 1 Joseph J Zilber School of Public Health, University of Wisconsin Milwaukee, PO Box 413, Milwaukee, WI 53201 USA; 2 Center for Global Health Equity, College of Nursing, University of Wisconsin Milwaukee, PO Box 413, Milwaukee, WI 53201-0413 USA; 3 CARE-International Malawi, CARE House, Kenyatta Road, Capital City, Lilongwe Malawi; 4 College of Nursing, University of Wisconsin Milwaukee, PO Box 413, Milwaukee, WI 53201-0413 USA; 5 Department of Economics, Chancellor College, University of Malawi, P.O. Box 280, Zomba, Malawi; 6 Professor Emeritus of Sociology, University of Pennsylvania and Visiting Scholar, California Center for Population Research, University of California-Los Angeles, 4284 Public Affairs Bldg, PO Box 957236, Los Angeles, CA 90095-7236 USA

**Keywords:** Quasi-experiment, Structural intervention, Food security, HIV, Malawi, Microfinance, Sustainable agriculture, Farmer field schools

## Abstract

The objective of the Savings, Agriculture, Governance, and Empowerment for Health (SAGE4Health) study was to evaluate the impact of a large-scale multi-level economic and food security intervention on health outcomes and HIV vulnerability in rural Malawi. The study employed a quasi-experimental non-equivalent control group design to compare intervention participants (n = 598) with people participating in unrelated programs in distinct but similar geographical areas (control, n = 301). We conducted participant interviews at baseline, 18–, and 36–months on HIV vulnerability and related health outcomes, food security, and economic vulnerability. Randomly selected households (n = 1002) were interviewed in the intervention and control areas at baseline and 36 months. Compared to the control group, the intervention led to increased HIV testing (OR 1.90; 95 % CI 1.29–2.78) and HIV case finding (OR = 2.13; 95 % CI 1.07–4.22); decreased food insecurity (OR = 0.74; 95 % CI 0.63–0.87), increased nutritional diversity, and improved economic resilience to shocks. Most effects were sustained over a 3-year period. Further, no significant differences in change were found over the 3-year study period on surveys of randomly selected households in the intervention and control areas. Although there were general trends toward improvement in the study area, only intervention participants’ outcomes were significantly better. Results indicate the intervention can improve economic and food security and HIV vulnerability through increased testing and case finding. Leveraging the resources of economic development NGOs to deliver locally-developed programs with scientific funding to conduct controlled evaluations has the potential to accelerate the scientific evidence base for the effects of economic development programs on health.

## Introduction

While it is specific behaviors that place most individuals at risk for HIV infection, two interrelated distal factors that appear to influence the HIV risk pathways are relative poverty and food insecurity [1, 2]. Interventions that break the cycle of poverty are of increasing interest to the development and public health communities given the potential for sustainable long-term impact. Our study [3, 4] examines the effects of a structural multi-level HIV intervention, referred to as SAFE, designed and implemented by a large non-governmental organization (NGO), CARE International, in rural Malawi, which has high levels of poverty, food insecurity, and HIV prevalence.

Microfinance or microlending [5] has the potential to help low income individuals access credit and become economically stable, which may result in improvements in health-related outcomes such as decreased intimate partner violence and unprotected sex [6, 7], lower pregnancy rates [8, 9], and decreased prevalence of sexually transmitted infections [10]. Village savings and loans (VS&L) group participation has the potential for impact along multiple causal pathways to HIV vulnerability [11–13]; however, inconsistent findings prompt the need for more evaluation [14–16].

Food insecurity directly impacts health and is associated with decreased adherence to HIV antiretroviral therapy [17, 18], incomplete virological suppression [17, 19], declines in physical health status [17, 20], worse immunological status [18, 21], increased incidence of serious illness [20], increased mortality [22], decreased survival [22], reduced condom use, and an increased occurrence of symptoms that may indicate sexually transmitted infections among sexually active women [23].

The CARE-designed Support to Able-Bodied Vulnerable Groups to Achieve Food Security (SAFE) intervention was structured to address this issue, primarily through teaching sustainable agriculture and crop diversification to improve dietary diversity and resilience toward drought and floods, and through the introduction of microfinance access. Microfinance programming was intended to allow program participants to purchase agricultural inputs at critical times, to promote income generating businesses, and to help ensure illness did not cause families to fall into a cycle of poverty.

In this paper, we present the main outcomes of the Savings, Agriculture, Governance, and Empowerment for Health (SAGE4Health) intervention study, including HIV vulnerability, food security, and economic stability. The primary study hypotheses were that the intervention group would improve relative to the comparison group on measures of HIV vulnerability, food security, and economic stability at the 18- and 36-month follow-ups.

## Methods

### Overview of SAGE4Health and the CARE Malawi SAFE Intervention

A comprehensive description of SAGE4Health methodology is described elsewhere [4]; a summary follows. SAGE4Health examines three interrelated samples designed to systematically evaluate the SAFE program, a community-based, structural, multilevel health and development intervention designed and implemented by CARE International-Malawi from January 2008–December 2010. Specifically, the intervention components focused on (1) improving farming practices and sustainable agriculture through implementation of Farmer Field Schools in over 9,000 households; (2) increasing access to savings and investment through formation of 443 Village Savings and Loans Groups (VS&L); (3) building capacity of local governance structures through trainings and meetings; and (4) integrating HIV education and gender empowerment into programs through training and education [24]. The SAFE program was funded primarily by the European Commission and partially by the Austrian Development Cooperation, whereas the research design and evaluation study reported herein were funded by the National Institute on Child Health and Human Development.

### Overview of the Study Design

To study the impact of the SAFE program, the SAGE4Health study examined three samples. First, we compared 598 SAFE households from three intervention geographical areas, called Traditional Authorities (TAs), to 301 “control” households residing in three non-intervention TAs matched on demographics and distance from an urban center. The control participants here were 301 households of children who were in schools where CARE was providing wholly different programming, programming that did not focus on food security or economic stability. In these schools, CARE was providing a brief HIV prevention program consisting primarily of prevention education for the children. Survey data was collected in three waves: baseline (during 2009), 18-month follow up and 36-month follow up. Second, a random community sample of 501 households within the intervention area and 501 in the control area was collected at baseline and 36-month follow up to check for threats to the internal validity of the evaluation as well as examine whether the intervention impacted community members who were not direct participants. The third sample consisted of 90 in-depth interviews and nine focus group discussions to gain a qualitative understanding of the intervention’s impact on participants.

### Study Setting

SAGE4Health was implemented in three intervention TAs (Njombwa, Kaomba, and Mwase) and three non-intervention TAs (Lukwa, Kawamba, and Chaima) within Kasungu District, located in rural west-central Malawi. Malawi’s population is young, rural and 72 % live below the international poverty line of US$1.25 per day [25, 26]. Among Malawian adults aged 15–49, approximately 10.0 % live with HIV [27]. The Malawi economy is dominated by the agriculture sector, which employs 80 % of the population, accounts for 42 % of national GDP, supplies 81 % of foreign exchange earnings and contributes significantly to national and household food security [28]. Aside from tobacco exports, Malawi’s economy is also highly dependent on foreign aid.

### SAGE4Health Procedures

A 23-module survey was used to capture a wide range of constructs of a social-ecological framework [29, 30] of HIV vulnerability that aligns with the multilevel nature of the SAFE intervention. Modules included questions focusing on economic status, food security, HIV risk behaviors, HIV testing behavior, sexually transmitted infection symptoms, gender empowerment, and gender-based violence. Anthropometric measurements were taken to quantitatively assess the nutritional status of study participants and children under 5 in the household.

The Malawian field research team administered the surveys face-to-face in Chichewa, the language spoken in the study area. Interview data were collected using Samsung Galaxy tablets utilizing the survey research platform Open Data Kit (https://opendatakit.org/). The password-protected data were uploaded daily from the tablets to a secure desktop computer.

## Assessment

### HIV Testing and Status Outcomes and HIV Risk Behavior

Self-reported HIV testing and status were collected at each assessment point. Self-reported HIV testing was a cumulative measure across time, as the question asked, “Have you ever been tested to see if you have the HIV virus?” Status was obtained by asking, “If you received your results, was the HIV test negative or positive?” To assess risk behavior, we used a standard behavioral frequency measure that included sexual activity with and without condoms and the number of sexual partners.

### Economic Outcomes

Instruments measuring economic factors were adapted from the Malawi Diffusion and Ideational Change Project (MDICP) led by Watkins et al [31] and previous CARE surveys. We assessed the frequency of events leading to economic crises, specifically a major illness or hospitalization; or an environmental disaster such as drought, flood, or fire within the last 12 months. We also asked respondents about their use of cash for the immediate expenses of coping with such shocks—whether it was a short-term emergency response such as selling crops or livestock, engaging in ganyu (working in another’s smallholder field for the short-term) [32], or receiving donations; or a more sustainable response such as relying on a loan from a VS&L group or one’s own savings. Given the important influence of ganyu, particularly in women headed households, on the rural Malawi economic landscape, we examined both adult and child participation in ganyu [33].

### Food Security Outcomes

To assess food security, we asked whether or not the respondent’s household had lacked enough food to meet the family’s needs for each of the previous 12 months (yes/no). The sum of the number of yes answers constitutes the variable “total months of food insecurity.”

Nutrition (i.e., dietary intake) was assessed with questions asked in a yes/no format about the type of foods consumed in the last three days, including vitamin A-rich vegetables like green leafy vegetables, yellow sweet potato, carrot, or pumpkin; other vegetables such as tomato, cucumber, onion; vitamin A-rich fruits like mango, papaya, malambe (baobob, an oval fruit with white powdery pulp), or masuku (a plum-sized wildfruit); or protein-rich foods like groundnuts. To gauge respondents’ response to food insecurity, questions were asked related to household strategies for coping when there was not enough food, such as engaging in ganyu or reducing the amount or number of meals. Physical measurements including height and weight were collected from children under the age of five to quantify malnutrition status. Body Mass Index (BMI  = weight/height^2^) and height-for-age (related to stunting) and weight-for-height (related to wasting) were calculated using the World Health Organization/National Center for Health Statistics/Centers for Disease Control and Prevention reference standards [34] for all children under 5. Nutritional status was expressed in relationship to the reference population (CDC 2000 reference population) in Z-scores. We used the WHO global database on child growth and malnutrition’s Z-score cut-off point of <−2 Standard deviation to classify moderate level low weight-for-age (WAZ), and ≤−3 standard deviation as severe levels. Percentages for malnutrition (moderate or severe malnutrition combined) were calculated for all participants at baseline, 18- and 36-month follow-ups.

## Sample Size

Statistical power was determined for the primary HIV sexual risk outcome and the primary food security outcomes. The sample size was 598 for the control group and 301 for the intervention group. Effect sizes (d) were calculated according to Cohen [35]. Based on generalized estimating equations (GEE) [36] and power calculation according to Lin and Myers [37], using a significance level of α = 0.05, power to detect a moderate difference (effect size = 0.5) is over 90 %.

## Statistical Methods

The analysis was based on longitudinal trajectory analysis comparing the intervention condition and control condition. First, descriptive statistics were calculated to present demographic variables between study conditions (i.e., intervention and control) at baseline. Differences between conditions were assessed using *t* tests for continuous variables and chi square analyses for categorical variables. Variables for which differences between intervention and control conditions were significant were treated as covariates when assessing the intervention effects.

Second, cross-tabulations were conducted to estimate the prevalence of primary outcome variables including HIV vulnerability (i.e., self-reported HIV testing, HIV infection), household economic crises and ganyu involvement, food security and dietary diversity and intake between intervention and control at baseline, 18-month follow-up and 36-month follow up.

Finally, the effectiveness of the intervention was investigated from the baseline to the 18-month assessment and from baseline to 36-month assessment, respectively. To assess intervention effects, the Generalized Estimating Equation (GEE) was adopted to control for repeated within-subject measurements. The GEE technique allows for a different number of observations on study participants at each assessment in the longitudinal study design. This model included a time-independent variable (study condition) as well as time-dependent variables (covariates and outcomes). The model was adjusted for the corresponding baseline measure for each outcome and other covariates to obtain adjusted odds ratios when the outcome was binary to assess the effect of the interventions. Count outcome was examined using Poisson distribution. The 95 % confidence intervals (CIs) around the adjusted ORs and the corresponding *p* values were also computed. For each model, we conducted the full model analysis with group by time interactions. Epi Info was used for analyzing children’s nutritional status, using CDC 2000 reference populations. All statistical analyses were performed using SAS, version 9.3 (SAS Institute Inc, Cary, NC).

## Results

### Participants

Of 598 participants allocated to the intervention condition, 564 (94.3 %) completed both the 18- and 36-month assessment. Of the 301 participants allocated to the control condition, 263 (87.4 %) completed both the 18- and 36-month assessment. Based on the number of households we approached and those who agreed to participate, the refusal rate was very low (0.41 %). There was no difference between intervention and comparison groups in refusal rate. No differences in attrition were observed between study conditions. No differences were observed in baseline variables for either study conditions in participants retained in the study compared with those unavailable for follow-up.

#### Intervention Implementation Fidelity

Intervention implementation fidelity checks were performed based on recommended treatment fidelity framework provided by the NIH’s Behavioral Change Consortium (BCC) [38].

First we examined whether there was treatment “contamination” between intervention and control participants. We found that no control participants reported receiving any components of the intervention (i.e., CARE-delivered farmer field school, VS&L), while all intervention participants reported receiving some elements of the intervention.

Second, in terms of satisfaction with the intervention services received, 94.5 % of intervention participants rated “very satisfied” with the CARE program. Over two thirds (67.9 %) of the intervention participants reported that “credit from CARE VS&L was sufficient to meet their household needs” while about one third (30.5 %) reported that “credit from CARE FFS was sufficient to meet their household needs.”

Third, when asked to identify the specific ways intervention benefited their households, more than half of the participants (55.8 %) reported that CARE VS&L benefited the household by assisting them to “start a business,” “pay health expenses” or “pay school fees.” In addition, 17.4 % of the participants reported that CARE FFS benefited them so they could “buy agriculture inputs.”

Finally, we examined exposure to any other related programs during the study period. Very few of those in the intervention or control groups participated in other non-CARE programs during the study that might be related to outcomes. For those participants who received a non-CARE agricultural education program from an extension provider, their participation did not differ by group (p = .089). Participation in the government subsidized fertilizer program was equivalent across groups as well (p = .298). Finally, participation in other credit services (such as a government program or local lenders) was equivalent across groups as well (p > .05).

#### Descriptive data

##### Participant Characteristics

Participants in the intervention group did not differ substantially from those in the control group on most demographic variables. However, there was evidence at baseline that participants in the intervention group were older (p = .040), had smaller household sizes (p = .001) and their households were less dominated by males (p = .039). There were no substantial baseline sociodemographic differences between community random samples in the intervention and control areas except for household size (p = .021) and marital status (p =.021; Table 1). See Weinhardt et al for more details. [4]

**Table 1:** Baseline characteristics of SAFE participant and community samples, by conditions (intervention vs. control)

Characteristics	SAFE participant sample		P value	Random community sample		P value
Demographics	SAFE intervention	Control group		SAFE intervention area	Control area	
	N = 598	N = 301		N = 501	N = 501	
Female participants (%)	398 (66.6)	201 (66.8)	.947	334 (66.7)	327 (65.5)	.704
Mean age of respondent in years (range)	40.4 (18–84)	38.5 (19–86)	.040	38.6 (17–84)	38.2 (3–98)	.658
Mean household size (range)	5.3 (1–11)	6.3 (2–14)	.001	4.6 (1–13)	4.9 (1 –12)	.021
Male head of household	495 (82.8)	265 (88.0)	.039	402 (80.2)	421 (84.2)	.101
Head of household literate	472 (78.9)	236 (78.4)	.856	375 (75.2)	363 (72.9)	.416
Marital status			.085			.021
Currently married/living together	492 (82.3)	261 (86.7)		385 (77.0)	404 (80.8)	
Separated	21 (3.5)	9 (3.0)		18 (3.6)	8 (1.6)	
Divorced	19 (3.2)	11 (3.7)		48 (9.6)	27 (5.4)	
Widowed	54 (9.0)	20 (6.6)		44 (8.8)	54 (10.8)	
Never married	12 (2.00)	0 (0)		5 (1.0)	7 (1.4)	
Education (highest level of school)			.122			.353
Primary	447 (74.7)	225 (74.8)		366 (73.2)	383 (76.9)	
Secondary	81 (13.5)	28 (9.3)		62 (12.4)	49 (9.8)	
University	0 (0)	0 (0)		0 (0)	1 (0.2)	
Other	2 (0.3)	1 (0.3)		0 (0)	0 (0)	
Never went to school	68 (11.4)	47 (15.6)		72 (14.4)	65 (13.1)	
Have multiple spouses (%)	68 (21.8)	41 (24.3)	.537	42 (16.7)	57 (22.5)	.097
No response	286	132		249	248	

#### Main Results

Table 2 displays the prevalence of the primary outcomes at baseline, 18-month, and 36-month follow-ups for the intervention and comparison conditions. Fourteen out of 15 indicators suggested differences between intervention and control groups in the expected directions.

**Table 2:** Prevalence of primary outcome indicators

	Expected change in intervention compared to control	Intervention			Control		
		Baseline	18 months	36 months	Baseline	18 months	36 months
Longitudinal data							
HIV vulnerability							
Reported HIV testing	Increase	292/564 (51.8 %)	400/563 (71.0 %)	368/492 (74.8 %)	169/263 (64.3 %)	179/263 (68.1 %)	145/200 (72.5 %)
Female reported HIV testing	Increase	200/373 (53.6 %)	277/373 (74.3 %)	239/315 (75.9 %)	117/172 (67.6 %)	125/173 (72.3 %)	93/119 (78.2 %)
Male reported HIV testing	Increase	92/191 (48.2 %)	123/190 (64.7 %)	129/177 (72.9 %)	52/90 (57.8 %)	54/90 (60.0 %)	52/81 (64.2 %)
Self-reported HIV-Positive status	Increase	16/564 (2.8 %)	32/564 (5.7 %)	33/564 (5.9 %)	9/263 (3.4 %)	10/263 (3.8 %)	9/263 (3.4 %)
Economic crisis and ganyu involvement							
Economic crises due to illness/hospitalization	Decrease	343/562 (61 %)	323/564 (57.3 %)	261/562 (46.4 %)	120/263 (45.6 %)	163/263 (62.0 %)	153/263 (58.2 %)
Economic crises due to environmental disaster	Decrease	88/559 (15.7 %)	16/563 (2.8 %)	20/562 (3.6 %)	12/263 (4.6 %)	7/263 (2.7 %)	11/263 (4.2 %)
Adult engaged in ganyu	Decrease	290/564 (51.4 %)	346/564 (61.3 %)	337/563 (59.9 %)	111/263 (42.2 %)	159/263 (60.5 %)	165/263 (62.7 %)
Child engaged in ganyu	Decrease	85/553 (15.4 %)	99/562 (17.6 %)	98/552 (17.8 %)	39/260 (15.0 %)	55/261 (21.1 %)	71/262 (27.1 %)
Food Security							
Household food security	Increase	165/564 (29.3 %)	309/564 (54.8 %)	308/531 (58.0 %)	71/262 (27.1 %)	117/263 (44.5 %)	129/245 (52.7 %)
Consuming vitamin A-rich vegetables	Increase	536/586 (95.0 %)	522/564 (92.6 %)	472/481 (98.1 %)	248/263 (94.3 %)	260/263 (98.9 %)	236/245 (96.3 %)
Consuming other vegetables	Increase	426/564 (75.5 %)	471/564 (83.5 %)	504/531 (94.9 %)	198/263 (75.3 %)	189/263 (71.9 %)	228/244 (93.4 %)
Consuming vitamin A-rich fruits	Increase	27/564 (4.8 %)	248/564 (44.0)	160/531 (30.1 %)	47/263 (17.9 %)	49/262 (18.7 %)	95/245 (38.8 %)
Consuming groundnuts	Increase	396/563 (70.3 %)	455/564 (80.7 %)	405/563 (71.9 %)	191/262 (72.9 %)	188/263 (71.5 %)	202/262 (77.1 %)
Reducing amount and number of meals to cope with food shortage	Decrease	59/398 (14.8 %)	15/252 (6.0 %)	15/197 (7.6 %)	17/191 (8.9 %)	17/146 (11.6 %)	7/116 (6.0 %)
Anthropometric measurements (malnutrition status of children)	Decrease	62/420 (14.8 %)	54/322 (16.8 %)	64/344 (18.6 %)	48/213 (22.5 %)	37/187 (19.8 %)	47/194 (24.2 %)

Effects of the intervention on the primary outcomes including HIV vulnerability, food security and dietary diversity, and economic security are presented in Table 3. These analyses were performed separately over the first 18-month period (baseline to 18-month assessment) and over the entire 36-month period (baseline to 36-month assessment). Below we describe major findings from the GEE analyses, adjusting for the corresponding baseline variables and covariates, for the major outcome domains of the study.

**Table 3:** Estimates of intervention effects on primary outcome indicators

Odds Ratio (95 % CI)					
	Expected change in intervention compared to control	Baseline to 18 months	P value	Baseline to 36 months	P value
HIV vulnerability					
Reported HIV testing	Increase	1.93 (1.45–2.58)	.001	1.90 (1.29–2.78)	<.001
Female reported HIV testing	Increase	2.00 (1.40–2.86)	<.001	1.70 (1.03–2.82)	.039
Male reported HIV testing	Increase	1.81 (1.10–2.99)	.019	2.12 (1.16–3.89)	.015
Self-reported HIV-Positive status	Increase	1.85 (1.09–3.14)	.023	2.13 (1.07–4.22)	.030
Economic crises and ganyu involvement					
Had economic crises due to illness/ hospitalization	Decrease	.44 (.31–.62)	<.001	.34 (.23–.50)	<.001
Had economic crises due to environmental disaster	Decrease	.27 (.09–.79)	.017	.22 (.08–.56)	.002
Adult engaged in ganyu	Decrease	.72 (.51–.99)	.049	.61 (.42–.87)	.007
Child engaged in ganyu	Decrease	.78 (.50–1.22)	.283	.55 (.34–.88)	.012
Food security					
Household food security (binary measure: yes vs. no)	Increase	1.36 (.93–1.97)	.108	1.12 (.75–1.67)	.585
Household food insecurity (actual counts of self-reported months (0–12) suffered from food insecurity)	Decrease	.79 (.64–.97)	.026	.74 (.63–.87)	<.001
Consuming vitamin A-rich vegetables	Increase	.12 (.03–.45)	.001	1.75 (.56–5.44)	.334
Consuming other vegetables	Increase	1.96 (1.27–3.02)	.002	1.29 (.64–2.60)	.479
Consuming vitamin A-rich fruits	Increase	14.8 (8.15–26.80)	<.001	2.92 (1.65–5.17)	<.001
Consuming groundnuts	Increase	1.90 (1.29–2.80)	.001	.87 (.58–1.29)	.482
Reducing amount and number of meals to cope with food Shortage	Decrease	.27 (.11–.67)	.005	.72 (.24–2.10)	.542
Anthropometric measurements (malnutrition status of children)	Decrease	1.52 (.80–2.90)	.205	1.27 (.54–3.01)	.585

**Table 4:** Primary outcome indicators for the random community samples (i.e., non-program participants from intervention area and control area) at baseline and 36-month

	Intervention area		Control area	
	Baseline	36-month	Baseline	36-month
HIV Vulnerability				
Reported HIV testing	64.7 % (323/499)	74.2 % (264/356)	58.6 % (291/497)	69.6 % (250/359)
Female reported HIV testing	64.8 % (215/332)	75.6 % (155/205)	59.1 % (191/323)	73.3 % (148/202)
Male reported HIV testing	64.7 % (108/167)	68.3 % (82/120)	58.1 % (100/172)	65.3 % (79/121)
Self-reported HIV-Positive status	4.2 % (21/501)	4.3 % (21/490)	2.0 % (10/501)	2.4 % (11/460)
Economic crisis and ganyu involvement				
Had economic crises due to illness/hospitalization	54.1 % (271/501)	54.0 % (301/557)	43.3 % (217/501)	43.7 % (221/506)
Had economic crises due to environmental disaster	2.0 % (10/501)	2.2 % (12/557)	3.2 % (16/501)	3.2 % (16/506)
Adult engaged in ganyu	54.2 % (271/500)	61 % (264/433)	50.1 % (251/501)	66.7 % (301/460)
Child engaged in ganyu	12.6 % (63/499)	14.3 % (68/477)	8.8 % (43/488)	14.9 % (67/451)
Food security				
Household food security	24.6 % (123/501)	41.4 % (189/456)	23.2 % (116/500)	37.2 % (165/444)
Consuming vitamin A-rich vegetables	94.2 % (472/501)	94.1 % (524/557)	91.6 % (459/501)	91.7 % (464/506)
Consuming other vegetables	52.3 % (262/501)	50.4 % (281/557)	44.5 % (223/501)	44.7 % (226/506)
Consuming vitamin A-rich fruits	13.2 % (66/501)	33.1 % (170/514)	47.7 % (239/501)	55.9 % (251/449)
Consuming groundnuts	59.9 % (300/501)	62.0 % (303/489)	58.9 % (295/501)	59.8 % (275/460)
Reducing amount and number of meals to cope with food shortage	17.9 % (68/379)	20.1 % (103/510)	25.7 % (98/382)	27.8 % (125/450)
Anthropometric measurements (malnutrition status of children)	18.7 % (49/262)	19.1 % (50/261)	20.9 % (78/373)	22.8 % (84/369)

## HIV Vulnerability

### Self-reported HIV Testing, HIV-Positive Status, and HIV Risk Behavior

Relative to participants in the control group, participants in the intervention group reported increased HIV testing between baseline and 18-month (adjusted OR = 1.93; 95 % CI 1.45–2.58) and between baseline and 36-month (adjusted OR = 1.90; 95 % CI 1.29–2.78). The effects hold true for both genders (18-month female adjusted OR = 2.0; 95 % CI 1.4–2.86; 18-month male adjusted OR = 1.81; 95 % CI 1.10–2.99). The intervention effects sustained for females (adjusted OR = 1.70; 95 % CI 1.03–2.82) and males (adjusted OR = 2.12; 95 % CI 1.16–3.89) at the 36-month follow up.

In addition to increased testing, participants in the intervention were more likely to report HIV-positive status at the 18-month follow-up assessment (adjusted OR = 1.85; 95 % CI 1.09–3.14) and over the entire 36-month period (adjusted OR = 2.13; 95 % CI 1.07–4.22) compared to baseline. We did not find intervention effects on HIV risk behavior, operationalized as unprotected sexual activity with multiple partners. Baseline levels of these behaviors were very low across all samples.

## Food Security

### Food Security and Dietary Diversity Outcomes

Participants in the intervention group were less likely than controls to report months of food insecurity (i.e., defined as the number of months in the past year with insufficient food) from baseline to 18-months (adjusted OR = .79; 95 % CI .64–.97) and to 36 months (adjusted OR = .74; 95 % CI .63–.87). Similarly, results of the GEE analyses suggest participants in the intervention group were more likely to report a significant increase in consumption of vitamin A-rich fruits (e.g., mango, papaya, malambe, masuku) (adjusted OR = 14.8; 95 % CI 8.15–26.8), other vegetables (adjusted OR = 1.96; 95 % CI 1.27–3.02) and groundnuts (adjusted OR = 1.90; 95 % CI 1.29–2.80) at 18-month follow up. Intervention effects were observed at the 36-month follow up for consumption of vitamin A-rich fruits only. Intervention households were also less likely than controls to reduce amount and number of meals to cope with food shortages at 18-months (adjusted OR = .27; 95 % CI .11–.67) but not at the 36-month follow up.

### Anthropometric Measurements, Stunting and Malnutrition

We did not find significant intervention effects on malnutrition or stunting based on anthropometric measures at 18 or 36 month follow ups (Table 3). There was also no time by group interaction in the randomly selected community samples on these measures.

## Economic Security

### Resilience to Economic Shocks and Dependence on Ganyu

Participants in the intervention group increased their resilience to economic crises. Relative to the control group, participants in the intervention group reported greater decreases in economic crises due to illness/hospitalization (adjusted OR = .44; 95 % CI .31–.62) from baseline to 18-month and from baseline to 36-month (adjusted OR = .34; 95 % CI .23–.50). Likewise, intervention participants reported greater decreases in economic crises due to environmental disaster from baseline to 18-month (adjusted OR .27; 95 % CI .10–.79) and from baseline to 36-months (adjusted OR = .22; 95 % CI .08–.56). Adults in the intervention group, relative to the control group, were less likely to start engaging in ganyu at 18-months (adjusted OR = .72; 95 % CI .51–.99), and sustained at 36-month follow up (adjusted OR = .61; 95 % CI .42–.87). Similarly, participant households in the intervention were less likely to report any engagement in child ganyu. This effect was detected at the 36-month follow up only.

### Randomly Selected Community Samples

We did not find significant group × time interaction effects among the randomly selected community sample on any of these measures (outcomes shown in Table 4). In other words, our data indicate these changes occurred in the intervention participant households and not in the surrounding households.

## Discussion

### Key Results

Our data suggest that this multi-level structural intervention addressing poverty and food security may have significant impacts on HIV vulnerability, as well as intended effects on economic stability and food security. Results were not uniformly positive, however, and indicate areas for improved and sustained efforts.

These findings, increased testing and known HIV-positive serostatus, are important and encouraging; people who are tested and know their serostatus are less likely to transmit HIV to others due to decreased risk behavior [39, 40], and decreased viral load if on ARV treatment, in addition to the personal health benefits of HIV treatment. This finding is particularly pertinent given that new treatment regimens enacted by Malawi in 2008 moved closer to universal ARV coverage–rather than waiting until CD4 cell (markers of immune function) count drops below 200 cells/mm^3^. Malawi was an early adopter of initiating ARV treatment once one’s CD4 count dropped to 350 or lower [41]. In addition, Malawi’s adoption of Option B+, the provision of ARVs to all pregnant women living with HIV for life, regardless of CD4 count, in late 2011, increased access. Likely as a result of these policies, Malawi saw a decrease in HIV incidence by 70 % across this time period [42]. Given Malawi’s commitment to initiating ARVs early among people living with HIV (PLHIV), it becomes all the more critical to increase HIV testing–ARVs cannot be initiated until one knows their serostatus. Because risk behavior did not increase across the SAGE4Health study period, it is likely that the increase in individuals reporting a HIV-positive serostatus is a result of the observed increased testing and therefore case-finding.

A policy environment supportive of providing HIV treatment is only one factor important to decrease HIV incidence—access to medications, and consistent food availability to take with medications, can be a major barrier. Alongside Malawi’s success, barriers to ARV initiation and adherence remain such as drug stock-outs, transportation costs to healthcare facilities, discrimination by healthcare providers against PLHIV, and food shortages [41]. The factor of nutrition is particularly acute among pregnant women on ARVs [43]; enhancing the need for combination structural interventions that together address HIV vulnerability and associated factors.

These data indicate that the CARE SAFE program contributed to improved dietary diversity and intake, providing evidence of food security important to HIV prevention and care. Malawi has a high prevalence of people living with micronutrient deficiencies, and, considering that some micronutrient deficiencies can contribute to adverse HIV treatment outcomes, dietary diversity becomes particularly relevant to PLHIV. Shivakoti et al. recently found that some, but not all, micronutrient deficiencies among treatment-naive PLHIV decreased after ARV-initiation [44]. This finding emphasizes the need for supplementation or increased dietary intake of a variety of foods supplying micronutrients. Intervention participants consumed increased levels of vitamin A-rich foods, which is not only beneficial to PLHIV–vitamin A is essential to address nutritional and growth needs, night blindness, proper functioning of the immune system, and due to its impact on the chance of survival of several causes of mortality such as diarrhea, malaria and pneumonia.

In addition to increased HIV-testing and case finding, and improved food security, the intervention also resulted in increased economic stability–another key pillar in HIV prevention. Intervention households were better able to absorb shocks without falling further into poverty, a likely impact of participation in VS&L groups. VS&L is an approach that allows community members increase their financial literacy, learn income-generating strategies, save money collectively, and to have an opportunity to borrow money from the group. The VS&L groups in this intervention also served as a platform to discuss and learn about HIV and other relevant social and community issues, an important VS&L design characteristic. [5] VS&L Associations have promoted a culture of saving and has provided loan accessibility to populations who would otherwise be unable to access them from banks or microfinance institutions. Microfinance’s impact in both poverty-alleviation and health is widely debated; current systematic reviews have found mixed evidence for microfinance’s ability to lift people out of poverty [45] and minimal evidence for impacting HIV-related outcomes. [14] However, VS&L Associations are different from larger microfinance programs, as they do not require external support; after initial training, they are completely self-run by group members. In a randomized controlled trial of CARE’s VS&L Associations in rural Malawi conducted by Innovations for Poverty Action around the same time period as the SAGE4Health Study, they found no increase in asset ownership and overall expenditures. However, they did find a small increase in savings balances and a small but significant increase in the use of savings to weather economic shocks–similar to our findings [46].

Participants in the intervention were also better able to respond to emergencies without resorting to ganyu, a livelihood strategy participants called “harvesting hunger” wherein they worked others’ fields while neglecting their own. Improving household resilience to shocks holds promise for reducing vulnerability to HIV and other hazards.

Despite positive intervention effects on related outcomes, we did not detect significant improvements on children’s anthropometric measures. This intervention may not be of sufficient strength to impact these outcomes or it could be that sustained intervention of this sort over a longer period of time, or longer follow up intervals, are necessary to result in significant, observable changes in malnutrition and stunting.

Although the aim of CARE’s economic development and food security intervention was not specifically HIV focused, there are plausible reasons why alleviating poverty and food insecurity could increase HIV testing. The pathways through which this occurs require further exploration, but may include increased testing access and healthcare-seeking behavior, women’s empowerment, or community support and cohesion. Further, addressing immediate economic and food security concerns may allow individuals to focus on more distal health threats. The CARE intervention seems to contribute to the promotion of HIV-testing and case-finding among participants and has potential to be adapted for similar resource-poor settings.

Recent research combining food security and microfinance approaches targeting have displayed promise; a pilot RCT in rural Kenya targeting PLHIV found improvements in health, food consumption, and food security [47]. This study adds to the body of research an example of an intervention designed to address persistent poverty, primarily through crop diversification designed to improve dietary diversity and resilience toward weather-related shocks; and access to credit and loans to purchase agricultural inputs at critical times, promote income-generating businesses, and to ensure illness does not lead families back into a cycle of poverty.

## Limitations

The major limitation in this study is the lack of randomization to study condition, and resulting potential selection bias. Conducting a non-randomized quasi-experiment is a reasonable choice for this type of field study in collaboration with an NGO delivering its own program to people and areas where they deem it most necessary. We were able to maximize external validity while taking steps to rule out threats to internal validity. In a quasi-experimental study of the effects of a large field-based intervention, it is important to rule out alternative explanations for observed intervention effects. In our study, the intervention and control groups consisted of people from demographically and geographically similar TAs, and the people in the intervention condition had chosen to be involved in the CARE SAFE program when it was offered to them.

Beyond controlling for baseline differences between intervention and control groups in all analyses, we wanted to ensure that changes occurring naturally in the areas during the time of the study were accounted for and did not explain what appear to be intervention effects. Our randomly-selected community samples, assessed at baseline and 36 months, showed no time by group interaction effects, indicating the two areas were experiencing the same historical trends during the study. Thus, we are confident that observed intervention effects among longitudinal participants are a result of the SAFE intervention and not due to changes occurring for other reasons in Malawi at the time the study was conducted.

We cannot rule out effects of self-selection bias based on the design of the intervention and its implementation. Individuals who chose to participate in the SAFE program may have had different motivations to improve their status at the beginning of the study than those who did not want to be involved. Those who participated could have been at higher risk and recognized they needed more help, or they could have been more confident they could complete the CARE program. Further, program staff or village leaders could have selectively encouraged people to participate. However, the samples were highly similar at baseline, demographically and on outcome measures.

## Generalizability

Although the SAFE program was designed with the current health and economic needs of the local Malawian communities targeted, we believe our findings are generalizable to other similar multi-level economic development programs conducted in low resource settings dependent on subsistence agriculture. We encourage additional rigorous evaluations of these and other programs in other geographic areas to increase the meager knowledge base on the health effects of economic development programs and structural HIV interventions.

## Interpretation

To our knowledge this is one of the first controlled studies to report effects of a large multi-level structural poverty reduction intervention, conducted independently by an NGO, on HIV vulnerability and other health outcomes. Although other consortiums (http://strive.lshtm.ac.uk/) and rigorous studies have examined structural interventions that include microfinance and gender empowerment training on health outcomes in sub Saharan Africa [7], to our knowledge, none have integrated microfinance and sustainable agriculture/food security interventions in the same package/model as a primary prevention approach to HIV. One recent study has used a similar combination of interventions to improve the health of PLHIV [47].

We are encouraged by these mixed findings—we found the intervention package designed and delivered by CARE had positive effects. Through this controlled study, we have identified areas in which it can be improved for greater impact. We also conclude there is great potential in this type of leveraged study design, in which highly skilled international NGOs with significant community engagement conduct their best, locally-tailored programs and partner with academia to conduct controlled evaluations of effects of multi-level programs in real-world settings.
